# 2,3;5,6-Di-*O*-isopropyl­idene-1-*O*-(2-phenyl­acet­yl)-α-d-mannofuran­ose

**DOI:** 10.1107/S1600536810032368

**Published:** 2010-08-18

**Authors:** Iulia A. Sacui, Peter Norris, Matthias Zeller

**Affiliations:** a Department of Chemistry, Youngstown State University, 1 University Plaza, Youngstown, OH 44555-3663, USA

## Abstract

The title compound, C_20_H_26_O_7_, was prepared by esterification of 2,3;5,6-di-*O*-isopropyl­idene-α-d-mannofuran­ose with phenyl­acetic acid under standard DCC/DMAP (DCC = dicyclohexylcarbodiimide and DMAP = 4-dimethylaminopyridine) con­ditions. The solid-state structure confirms the retention of the α-configuration at the anomeric C atom. The compound is characterized by a relatively rigid framework with only a few degrees of freedom. Comparison with other di-*O*-isopropyl­idenemannofuran­ose derivatives shows the main differences to be associated with the flexible dimethyl­dioxolane ring, and that there are only small differences for the 2,3-*O*-isopropyl­idene-α-d-manno­furan­ose backbone. The packing is marked by a large number of weak C—H⋯O inter­actions.

## Related literature

For general background, see: Sacui *et al.* (2008 ▶). For related structures, see: Aebischer *et al.* (1982 ▶); Dang *et al.* (2001 ▶); Miner *et al.* (2004 ▶); Sheldrick *et al.* (1985 ▶); Zhao *et al.* (2006 ▶). For details of the Cambridge Structural Database, see: Allen (2002 ▶).
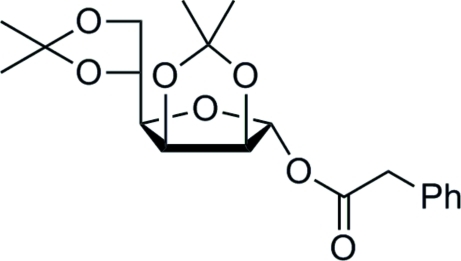


## Experimental

### 

#### Crystal data

C_20_H_26_O_7_
*M* *_r_* = 378.41
Orthorhombic, 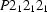

*a* = 5.6174 (3) Å
*b* = 13.0946 (8) Å
*c* = 25.2021 (15) Å
*V* = 1853.81 (19) Å^3^
*Z* = 4
Mo *K*α radiation
μ = 0.10 mm^−1^
*T* = 100 K
0.60 × 0.33 × 0.12 mm

#### Data collection

Bruker SMART APEX CCD diffractometer
Absorption correction: multi-scan (*SADABS* in *SAINT-Plus*; Bruker, 2003 ▶) *T*_min_ = 0.812, *T*_max_ = 0.988
18953 measured reflections
2668 independent reflections
2652 reflections with *I* > 2σ(*I*)
*R*_int_ = 0.040

#### Refinement

*R*[*F* ^2^ > 2σ(*F* ^2^)] = 0.051
*wR*(*F* ^2^) = 0.117
*S* = 1.33
2668 reflections
248 parameters
H-atom parameters constrained
Δρ_max_ = 0.37 e Å^−3^
Δρ_min_ = −0.26 e Å^−3^

### 

Data collection: *SMART for WNT/2000* (Bruker, 2002 ▶); cell refinement: *SAINT-Plus* (Bruker, 2003 ▶); data reduction: *SAINT-Plus*; program(s) used to solve structure: *SHELXTL* (Sheldrick, 2008 ▶); program(s) used to refine structure: *SHELXTL*; molecular graphics: *SHELXTL* and *Mercury* (Macrae *et al.*, 2008 ▶); software used to prepare material for publication: *SHELXTL* and *publCIF* (Westrip, 2010 ▶).

## Supplementary Material

Crystal structure: contains datablocks global, I. DOI: 10.1107/S1600536810032368/bv2157sup1.cif

Structure factors: contains datablocks I. DOI: 10.1107/S1600536810032368/bv2157Isup2.hkl

Additional supplementary materials:  crystallographic information; 3D view; checkCIF report

## Figures and Tables

**Table 1:** Hydrogen-bond geometry (Å, °)

*D*—H⋯*A*	*D*—H	H⋯*A*	*D*⋯*A*	*D*—H⋯*A*
C1—H1⋯O6^i^	1.00	2.46	3.382 (3)	153
C17—H17⋯O5^ii^	0.95	2.54	3.483 (3)	172
C2—H2⋯O7^iii^	1.00	2.65	3.591 (3)	156
C3—H3⋯O1^iii^	1.00	2.67	3.559 (3)	148
C8—H8*C*⋯O1^iii^	0.98	2.62	3.570 (3)	164
C12—H12*A*⋯O7^iv^	0.98	2.70	3.323 (4)	122

Symmetry codes: (i) 
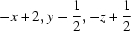
; (ii) 
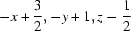
; (iii) 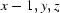; (iv) 
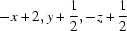
.

**Fig. 1.**
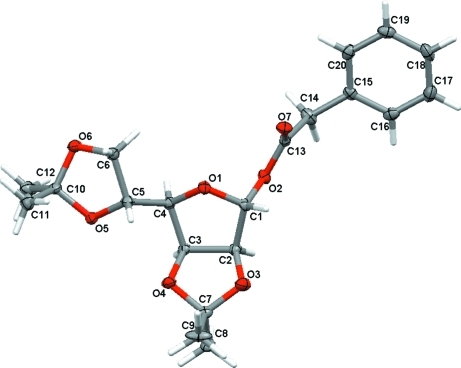
Molecular structure showing 50% probability displacement ellipsoids.

**Fig. 2.**
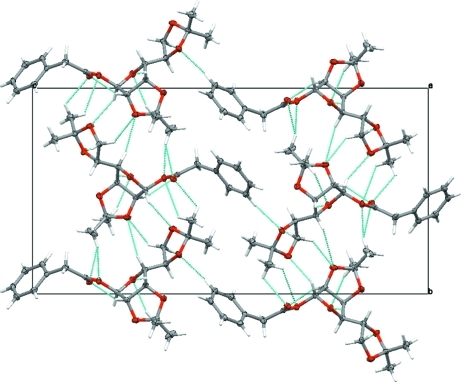
Molecular packing and intermolecular interactions. C—H···O interactions are symbolized by blue dashed lines. View down the a axis.

**Fig. 3.**
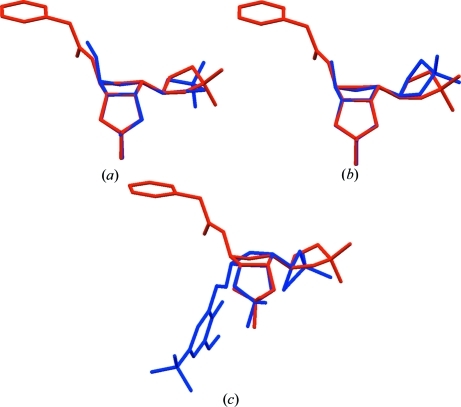
Least square overlays of the title compound with (a) 2,3:5,6-di-O-isopropylidene-α-D-mannofuranose (CUFYIL, Sheldrick et al., 1985; Miner et al., 2004), (b) methyl 2,3:5,6-di-O-isopropylidene-β-L-gulofuranoside (BOXQAG, Dang et al., 2001), (c) 5-tert-butyl-3-((6-(2,2-dimethyl-1,3-dioxolan-4-yl)-2,2-dimethyltetrahydrofuro(3,4-d)(1,3)dioxol-4-yloxy)methyl)-2-hydroxybenzaldehyde (QEMKID, Zhao et al., 2006).

## supplementary crystallographic information

### Comment

The title compound, 1-*O*-(2-phenylacetyl)-2,3;5,6-di-*O*-isopropylidene-α-D-mannofuranose, was prepared as part of our ongoing work on the synthesis and metal-catalyzed decomposition of carbohydrate-derived diazo compounds (Sacui *et al.*, 2008). It was prepared by esterification of commercially available 2,3;5,6-di-*O*-isopropylidene-α-D-mannofuranose with phenylacetic acid under standard DCC/DMAP (DCC = dicyclohexylcarbodiimide and DMAP = 4-dimethylaminopyridine) conditions. From ^1^H NMR data, the precursor lactol (before esterification) exists in solution only as the α-anomer, which is in agreement with the reported solid state structure (Sheldrick *et al.*, 1985; Miner *et al.*, 2004). ^1^H NMR data point in the same direction for the title compound. To obtain proof of the configuration of the title compound in the solid state we investigated its structure by single-crystal diffraction.

Slow evaporation of a solution of the compound in 95% ethyl alcohol led to formation of crystals of the title compound that could be analyzed by single-crystal diffraction. The compound crystallizes in an orthorhombic setting in *P*2_1_2_1_2_1_ with one crystallographically independent molecule. The solid state structure confirms retention of the α-configuration at C-1 of the furanose ring under the conditions of the DCC-promoted esterification.

No strong directional intermolecular interactions are present in the structure of the title compound. For the carbohydrate part of the molecules the packing is instead marked by a large number of weaker C—H···O interactions as depicted in Fig. 2 (for numerical values, see Table 1). The phenyl rings are not involved in any π–π stacking interactions but instead they do group with the isopropylidene moieties to form layers perpendicular to the (110) plane characterized by the absence of any directional forces. Thus a semi-layered structure is created with alternating sections dominated by C—H···O interactions (the carbohydrate layers) and layers with only van der Waals / dispersion forces (the phenyl-isopropylidene layers).

All bond lengths and angles in the title compound are within the expected ranges for an organic compound. For the dihedral angles, most are fixed by the rigid backbone of the isopropylidene-mannofuranose skeleton, and the molecule exhibits only a few selected degrees of freedom. This becomes clearly apparent when, for example, comparing the title compound with other related compounds with a 2,3;5,6-di-*O*-isopropylidene-mannofuranose skeleton. A search in the Cambridge Crystallographic Database (version 5.31, 2010; Allen, 2002) revealed four related compounds, one of which has an *L*-mannofuranose sugar (BIRFUD, Aebischer *et al.*, 1982), and one a β connection at carbon C1 (QEMKID, Zhao *et al.*, 2006). The other two compounds are the lactol itself (CUFYIL, Sheldrick *et al.*, 1985; Miner *et al.*, 2004), and the 1-*O*-methyl derivative of the title compound (BOXQAG, Dang *et al.*, 2001). Least square overlays of the latter two with the title compound are shown in Figs. 3a and 3b (title compound in red). The 2,3-*O*-isopropylidene-mannofuranose skeleton in all three compounds shows only small deviations and virtually all conformational differences are limited to the more flexible second isopropylidene moiety, which is rotated differently when compared to the title compound and which also exhibits different types of envelope conformations: In the title compound the isopropylidene unit is out of plane with the other four atoms constituting the five membered ring. In the methyl derivative it is the C—H group, and in the lactol, the methylene group, thus confirming that the energies of the different conformers of the dimethyldioxolane rings are indeed very close. For compound QEMKID (Zhao *et al.*, 2006), which features a β connection at carbon C1, a different conformation is also found for the actual sugar with C1 and O1 changing their roles in the five membered ring (Figure 3c). Overall, the crystal structure of the title compound provides evidence that appending an ester group at C-1 does little to alter the conformation of the mannofuranose ring.

### Experimental

The title compound was prepared from 1,2;5,6-di-*O*-isopropylidene-α-D-mannofuranose by esterification with phenylacetic acid using standard DCC/DMAP (DCC = dicyclohexylcarbodiimide and DMAP = 4-dimethylaminopyridine) conditions in a similar fashion to that described earlier (Sacui *et al.*, 2008). A colorless solid was obtained after column chromatography (silica gel, 4:1 hexanes–EtOAc). Crystallization from 95% ethyl alcohol yielded colourless crystals suitable for single-crystal X-ray diffraction.

### Refinement

Treatment of hydrogen atoms: Hydrogen atoms have been added in calculated positions with C—H bond lengths between 0.95 and 1.00 Å and have been refined with an isotropic displacement parameter of 1.5 times (CH_3_) or 1.2 times (C—H and CH_2_) that of the equivalent isotropic displacement parameter of the adjacent carbon atom. Methyl H atoms were allowed to rotate to best fit the experimental electron density.

Assignment of absolute structure: Friedel pairs have been merged before refinement. The absolute structure assignment is based on the known configuration of carbon atoms retaining their configuration during the synthesis.

### Crystal data

**Table:**

C_20_H_26_O_7_	*F*(000) = 808
*M**_r_* = 378.41	*D*_x_ = 1.356 Mg m^−^^3^
Orthorhombic, *P*2_1_2_1_2_1_	Mo *K*α radiation, λ = 0.71073 Å
Hall symbol: P 2ac 2ab	Cell parameters from 9604 reflections
*a* = 5.6174 (3) Å	θ = 2.9–30.4°
*b* = 13.0946 (8) Å	µ = 0.10 mm^−^^1^
*c* = 25.2021 (15) Å	*T* = 100 K
*V* = 1853.81 (19) Å^3^	Block, colourless
*Z* = 4	0.60 × 0.33 × 0.12 mm

### Data collection

**Table:**

Bruker SMART APEX CCD diffractometer	2668 independent reflections
Radiation source: fine-focus sealed tube	2652 reflections with *I* > 2σ(*I*)
graphite	*R*_int_ = 0.040
ω scans	θ_max_ = 28.3°, θ_min_ = 1.6°
Absorption correction: multi-scan (*SADABS* in *SAINT-Plus*; Bruker, 2003)	*h* = −7→7
*T*_min_ = 0.812, *T*_max_ = 0.988	*k* = −17→17
18953 measured reflections	*l* = −32→33

### Refinement

**Table:**

Refinement on *F*^2^	Primary atom site location: structure-invariant direct methods
Least-squares matrix: full	Secondary atom site location: difference Fourier map
*R*[*F*^2^ > 2σ(*F*^2^)] = 0.051	Hydrogen site location: inferred from neighbouring sites
*wR*(*F*^2^) = 0.117	H-atom parameters constrained
*S* = 1.33	*w* = 1/[σ^2^(*F*_o_^2^) + (0.0345*P*)^2^ + 1.2578*P*] where *P* = (*F*_o_^2^ + 2*F*_c_^2^)/3
2668 reflections	(Δ/σ)_max_ < 0.001
248 parameters	Δρ_max_ = 0.37 e Å^−^^3^
0 restraints	Δρ_min_ = −0.26 e Å^−^^3^

### Special details

**Table:**

Geometry. All e.s.d.'s (except the e.s.d. in the dihedral angle between two l.s. planes) are estimated using the full covariance matrix. The cell e.s.d.'s are taken into account individually in the estimation of e.s.d.'s in distances, angles and torsion angles; correlations between e.s.d.'s in cell parameters are only used when they are defined by crystal symmetry. An approximate (isotropic) treatment of cell e.s.d.'s is used for estimating e.s.d.'s involving l.s. planes.
Refinement. Refinement of *F*^2^ against ALL reflections. The weighted *R*-factor *wR* and goodness of fit *S* are based on *F*^2^, conventional *R*-factors *R* are based on *F*, with *F* set to zero for negative *F*^2^. The threshold expression of *F*^2^ > σ(*F*^2^) is used only for calculating *R*-factors(gt) *etc*. and is not relevant to the choice of reflections for refinement. *R*-factors based on *F*^2^ are statistically about twice as large as those based on *F*, and *R*-factors based on ALL data will be even larger.

### Fractional atomic coordinates and isotropic or equivalent isotropic displacement parameters (Å^2^)

**Table:**

	*x*	*y*	*z*	*U*_iso_*/*U*_eq_	
O1	0.9107 (3)	0.56927 (14)	0.25276 (7)	0.0161 (4)	
O6	0.9344 (4)	0.81639 (14)	0.35444 (8)	0.0199 (4)	
O7	1.1329 (3)	0.56059 (15)	0.14428 (7)	0.0193 (4)	
O2	0.7450 (3)	0.56285 (14)	0.16757 (7)	0.0167 (4)	
O4	0.5450 (4)	0.47655 (14)	0.33085 (7)	0.0200 (4)	
O3	0.5940 (4)	0.37204 (14)	0.26113 (8)	0.0230 (4)	
O5	0.6671 (3)	0.69122 (14)	0.36960 (7)	0.0184 (4)	
C5	0.8432 (5)	0.6452 (2)	0.33622 (10)	0.0166 (5)	
H5	0.9090	0.5824	0.3534	0.020*	
C13	0.9301 (5)	0.57991 (19)	0.13408 (10)	0.0155 (5)	
C3	0.5230 (5)	0.54195 (19)	0.28614 (9)	0.0140 (5)	
H3	0.3639	0.5761	0.2846	0.017*	
C18	1.2659 (6)	0.5338 (2)	−0.04967 (10)	0.0250 (6)	
H18	1.3616	0.5120	−0.0786	0.030*	
C17	1.0503 (6)	0.4865 (2)	−0.03966 (11)	0.0257 (6)	
H17	0.9964	0.4330	−0.0621	0.031*	
C4	0.7276 (5)	0.61858 (19)	0.28397 (9)	0.0147 (5)	
H4	0.6738	0.6824	0.2657	0.018*	
C16	0.9133 (5)	0.5172 (2)	0.00314 (11)	0.0208 (6)	
H16	0.7668	0.4836	0.0102	0.025*	
C10	0.7721 (5)	0.77973 (19)	0.39329 (10)	0.0167 (5)	
C2	0.5653 (5)	0.47073 (19)	0.23826 (10)	0.0158 (5)	
H2	0.4323	0.4733	0.2119	0.019*	
C15	0.9879 (5)	0.59677 (19)	0.03595 (9)	0.0162 (5)	
C12	0.5801 (6)	0.8580 (2)	0.40282 (12)	0.0248 (6)	
H12A	0.6512	0.9201	0.4176	0.037*	
H12B	0.4629	0.8305	0.4278	0.037*	
H12C	0.5013	0.8743	0.3692	0.037*	
C20	1.2031 (5)	0.6441 (2)	0.02492 (10)	0.0192 (5)	
H20	1.2561	0.6986	0.0468	0.023*	
C8	0.2243 (5)	0.3600 (2)	0.31076 (11)	0.0183 (5)	
H8A	0.1581	0.3657	0.3466	0.028*	
H8B	0.1902	0.2920	0.2964	0.028*	
H8C	0.1520	0.4120	0.2879	0.028*	
C1	0.8021 (4)	0.50643 (19)	0.21488 (9)	0.0145 (5)	
H1	0.9061	0.4467	0.2063	0.017*	
C6	1.0386 (5)	0.72767 (19)	0.33117 (10)	0.0178 (5)	
H6A	1.1843	0.7072	0.3505	0.021*	
H6B	1.0795	0.7399	0.2935	0.021*	
C9	0.6133 (5)	0.3004 (3)	0.34852 (14)	0.0324 (8)	
H9A	0.7855	0.3125	0.3482	0.049*	
H9B	0.5804	0.2310	0.3359	0.049*	
H9C	0.5528	0.3081	0.3848	0.049*	
C19	1.3418 (5)	0.6130 (2)	−0.01758 (11)	0.0225 (6)	
H19	1.4890	0.6461	−0.0247	0.027*	
C7	0.4927 (5)	0.3763 (2)	0.31284 (10)	0.0176 (5)	
C11	0.9020 (6)	0.7498 (2)	0.44388 (11)	0.0244 (6)	
H11A	1.0245	0.6989	0.4356	0.037*	
H11B	0.7882	0.7208	0.4692	0.037*	
H11C	0.9770	0.8104	0.4594	0.037*	
C14	0.8400 (5)	0.6279 (2)	0.08310 (10)	0.0215 (6)	
H14A	0.6727	0.6071	0.0772	0.026*	
H14B	0.8436	0.7032	0.0866	0.026*	

### Atomic displacement parameters (Å^2^)

**Table:**

	*U*^11^	*U*^22^	*U*^33^	*U*^12^	*U*^13^	*U*^23^
O1	0.0129 (8)	0.0187 (9)	0.0166 (8)	−0.0001 (8)	0.0006 (7)	−0.0021 (7)
O6	0.0192 (9)	0.0144 (8)	0.0259 (9)	−0.0018 (8)	0.0071 (8)	−0.0006 (7)
O7	0.0156 (9)	0.0206 (9)	0.0216 (9)	−0.0011 (8)	0.0021 (8)	0.0027 (8)
O2	0.0154 (9)	0.0201 (8)	0.0145 (8)	0.0026 (8)	0.0005 (7)	0.0000 (7)
O4	0.0233 (10)	0.0199 (9)	0.0169 (8)	−0.0102 (8)	−0.0028 (8)	0.0029 (7)
O3	0.0250 (10)	0.0139 (9)	0.0300 (10)	0.0024 (8)	0.0110 (9)	0.0008 (8)
O5	0.0172 (9)	0.0199 (9)	0.0180 (8)	−0.0060 (8)	0.0029 (8)	−0.0050 (7)
C5	0.0160 (12)	0.0179 (11)	0.0160 (11)	−0.0028 (11)	0.0003 (10)	0.0013 (9)
C13	0.0192 (12)	0.0127 (11)	0.0146 (11)	0.0018 (10)	0.0006 (10)	−0.0026 (9)
C3	0.0128 (11)	0.0145 (11)	0.0148 (10)	−0.0010 (9)	−0.0007 (9)	−0.0001 (9)
C18	0.0350 (16)	0.0256 (13)	0.0143 (11)	0.0100 (13)	0.0066 (12)	0.0025 (10)
C17	0.0376 (17)	0.0194 (12)	0.0200 (12)	0.0000 (13)	−0.0058 (12)	−0.0029 (10)
C4	0.0152 (11)	0.0136 (10)	0.0152 (10)	0.0009 (10)	0.0001 (10)	−0.0002 (9)
C16	0.0208 (13)	0.0176 (12)	0.0240 (13)	−0.0025 (11)	−0.0035 (11)	0.0054 (10)
C10	0.0165 (12)	0.0148 (11)	0.0187 (11)	−0.0045 (10)	0.0022 (10)	−0.0016 (9)
C2	0.0137 (11)	0.0157 (11)	0.0181 (11)	−0.0010 (10)	0.0008 (10)	−0.0020 (9)
C15	0.0179 (12)	0.0166 (11)	0.0141 (11)	0.0042 (10)	−0.0010 (10)	0.0049 (9)
C12	0.0226 (14)	0.0208 (12)	0.0309 (14)	0.0019 (12)	0.0099 (12)	0.0019 (11)
C20	0.0222 (13)	0.0156 (11)	0.0196 (11)	0.0017 (11)	−0.0039 (11)	−0.0009 (10)
C8	0.0127 (11)	0.0183 (12)	0.0239 (12)	−0.0008 (10)	0.0009 (10)	−0.0026 (10)
C1	0.0121 (11)	0.0157 (11)	0.0157 (10)	0.0018 (10)	0.0027 (9)	−0.0005 (9)
C6	0.0168 (12)	0.0165 (11)	0.0202 (11)	−0.0016 (10)	0.0022 (10)	−0.0027 (10)
C9	0.0131 (13)	0.0340 (16)	0.0501 (19)	−0.0029 (13)	−0.0004 (13)	0.0234 (15)
C19	0.0187 (13)	0.0227 (13)	0.0262 (13)	0.0025 (12)	0.0027 (11)	0.0076 (11)
C7	0.0130 (11)	0.0175 (12)	0.0223 (12)	0.0000 (10)	−0.0003 (10)	0.0023 (10)
C11	0.0241 (14)	0.0281 (14)	0.0209 (12)	−0.0015 (13)	−0.0035 (12)	−0.0045 (11)
C14	0.0211 (13)	0.0251 (13)	0.0183 (12)	0.0079 (12)	0.0028 (11)	0.0039 (10)

### Geometric parameters (Å, °)

**Table:**

O1—C1	1.400 (3)	C10—C12	1.507 (4)
O1—C4	1.447 (3)	C10—C11	1.520 (4)
O6—C10	1.421 (3)	C2—C1	1.528 (3)
O6—C6	1.427 (3)	C2—H2	1.0000
O7—C13	1.195 (3)	C15—C20	1.386 (4)
O2—C13	1.358 (3)	C15—C14	1.506 (4)
O2—C1	1.439 (3)	C12—H12A	0.9800
O4—C7	1.420 (3)	C12—H12B	0.9800
O4—C3	1.421 (3)	C12—H12C	0.9800
O3—C7	1.423 (3)	C20—C19	1.386 (4)
O3—C2	1.424 (3)	C20—H20	0.9500
O5—C10	1.431 (3)	C8—C7	1.523 (4)
O5—C5	1.432 (3)	C8—H8A	0.9800
C5—C4	1.509 (3)	C8—H8B	0.9800
C5—C6	1.545 (4)	C8—H8C	0.9800
C5—H5	1.0000	C1—H1	1.0000
C13—C14	1.517 (3)	C6—H6A	0.9900
C3—C4	1.527 (4)	C6—H6B	0.9900
C3—C2	1.543 (3)	C9—C7	1.502 (4)
C3—H3	1.0000	C9—H9A	0.9800
C18—C19	1.382 (4)	C9—H9B	0.9800
C18—C17	1.384 (5)	C9—H9C	0.9800
C18—H18	0.9500	C19—H19	0.9500
C17—C16	1.385 (4)	C11—H11A	0.9800
C17—H17	0.9500	C11—H11B	0.9800
C4—H4	1.0000	C11—H11C	0.9800
C16—C15	1.395 (4)	C14—H14A	0.9900
C16—H16	0.9500	C14—H14B	0.9900
			
C1—O1—C4	108.84 (19)	H12A—C12—H12B	109.5
C10—O6—C6	105.74 (19)	C10—C12—H12C	109.5
C13—O2—C1	115.40 (19)	H12A—C12—H12C	109.5
C7—O4—C3	106.61 (18)	H12B—C12—H12C	109.5
C7—O3—C2	106.85 (19)	C19—C20—C15	121.0 (3)
C10—O5—C5	107.5 (2)	C19—C20—H20	119.5
O5—C5—C4	108.2 (2)	C15—C20—H20	119.5
O5—C5—C6	104.2 (2)	C7—C8—H8A	109.5
C4—C5—C6	113.3 (2)	C7—C8—H8B	109.5
O5—C5—H5	110.3	H8A—C8—H8B	109.5
C4—C5—H5	110.3	C7—C8—H8C	109.5
C6—C5—H5	110.3	H8A—C8—H8C	109.5
O7—C13—O2	124.2 (2)	H8B—C8—H8C	109.5
O7—C13—C14	126.0 (2)	O1—C1—O2	111.1 (2)
O2—C13—C14	109.8 (2)	O1—C1—C2	107.23 (19)
O4—C3—C4	111.0 (2)	O2—C1—C2	106.4 (2)
O4—C3—C2	104.02 (19)	O1—C1—H1	110.6
C4—C3—C2	104.7 (2)	O2—C1—H1	110.6
O4—C3—H3	112.2	C2—C1—H1	110.6
C4—C3—H3	112.2	O6—C6—C5	104.1 (2)
C2—C3—H3	112.2	O6—C6—H6A	110.9
C19—C18—C17	120.0 (3)	C5—C6—H6A	110.9
C19—C18—H18	120.0	O6—C6—H6B	110.9
C17—C18—H18	120.0	C5—C6—H6B	110.9
C18—C17—C16	119.9 (3)	H6A—C6—H6B	109.0
C18—C17—H17	120.1	C7—C9—H9A	109.5
C16—C17—H17	120.1	C7—C9—H9B	109.5
O1—C4—C5	105.8 (2)	H9A—C9—H9B	109.5
O1—C4—C3	105.15 (19)	C7—C9—H9C	109.5
C5—C4—C3	116.4 (2)	H9A—C9—H9C	109.5
O1—C4—H4	109.8	H9B—C9—H9C	109.5
C5—C4—H4	109.8	C18—C19—C20	119.9 (3)
C3—C4—H4	109.8	C18—C19—H19	120.0
C17—C16—C15	120.8 (3)	C20—C19—H19	120.0
C17—C16—H16	119.6	O4—C7—O3	104.2 (2)
C15—C16—H16	119.6	O4—C7—C9	109.1 (2)
O6—C10—O5	104.52 (19)	O3—C7—C9	110.0 (2)
O6—C10—C12	109.8 (2)	O4—C7—C8	110.2 (2)
O5—C10—C12	108.8 (2)	O3—C7—C8	111.0 (2)
O6—C10—C11	110.9 (2)	C9—C7—C8	112.0 (2)
O5—C10—C11	109.8 (2)	C10—C11—H11A	109.5
C12—C10—C11	112.7 (2)	C10—C11—H11B	109.5
O3—C2—C1	109.6 (2)	H11A—C11—H11B	109.5
O3—C2—C3	104.44 (19)	C10—C11—H11C	109.5
C1—C2—C3	104.5 (2)	H11A—C11—H11C	109.5
O3—C2—H2	112.6	H11B—C11—H11C	109.5
C1—C2—H2	112.6	C15—C14—C13	111.8 (2)
C3—C2—H2	112.6	C15—C14—H14A	109.3
C20—C15—C16	118.5 (2)	C13—C14—H14A	109.3
C20—C15—C14	121.2 (2)	C15—C14—H14B	109.3
C16—C15—C14	120.3 (3)	C13—C14—H14B	109.3
C10—C12—H12A	109.5	H14A—C14—H14B	107.9
C10—C12—H12B	109.5		

### Hydrogen-bond geometry (Å, °)

**Table:**

*D*—H···*A*	*D*—H	H···*A*	*D*···*A*	*D*—H···*A*
C1—H1···O6^i^	1.00	2.46	3.382 (3)	153.
C17—H17···O5^ii^	0.95	2.54	3.483 (3)	172.
C2—H2···O7^iii^	1.00	2.65	3.591 (3)	156.
C3—H3···O1^iii^	1.00	2.67	3.559 (3)	148.
C8—H8C···O1^iii^	0.98	2.62	3.570 (3)	164.
C12—H12A···O7^iv^	0.98	2.70	3.323 (4)	122.

Symmetry codes: (i) −*x*+2, *y*−1/2, −*z*+1/2; (ii) −*x*+3/2, −*y*+1, *z*−1/2; (iii) *x*−1, *y*, *z*; (iv) −*x*+2, *y*+1/2, −*z*+1/2.
